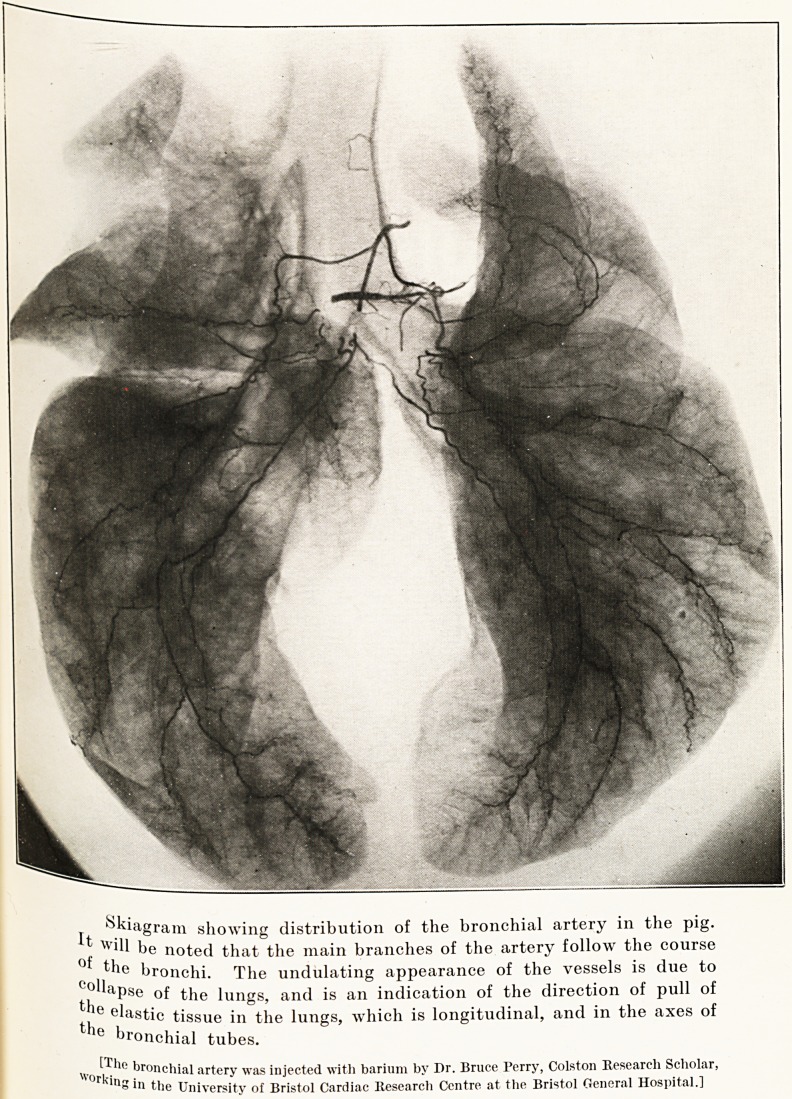# Problems in the Treatment of Hæmoptysis

**Published:** 1928

**Authors:** Hugh H. Carleton

**Affiliations:** Assistant Physician, Bristol General Hospital; Consulting Physician, Bristol Eye Hospital


### PROBLEMS IN THE TREATMENT OF HÆMOPTYSIS.

BY

Hugh H. Carleton, M.A., M.D., M.R.C.P.,

*Assistant Physician, Bristol General Hospital;*
*Consulting Physician, Bristol Eye Hospital.*

---

It must be taken as an axiom that two conditions are necessary for the occurrence of hæmoptysis. In the first place, there must be the rupture of a blood-vessel; secondly, the ruptured vessel must burst into some part of the bronchial tree. The passage of red blood corpuscles by diapedesis through the capillaries of congested lung tissue in pneumonia gives rise to blood-stained sputum, but not to true hæmoptysis. The tuberculous process in its earliest beginnings is a proliferative one, filling, or replacing, completely the tissues in which it arises, and there is, moreover, a marked tendency for the early tubercle to obliterate blood-vessels as it advances. At this stage the tissues are closely packed with the products of the tuberculous process. There is no space into which the congested pulmonary vessels can bleed to any serious extent. An initial hæmoptysis occurring at this stage can never be great in amount, and while it may be of the highest importance diagnostically, it presents little or no danger to the patient. True initial hæmoptysis rarely exceeds one or two drachms in amount.

As the tuberculous process advances and caseation occurs, sooner or later some degree of liquefaction of the caseous material begins, and the products of disintegration are coughed up, leaving a cavity which may be extremely small, but, nevertheless, the conditions necessary for a more extensive hæmoptysis are now established. A cavity must be in direct communication with the airway of the bronchial tree. The tuberculous focus is now no longer tightly packed, and if a vessel is eroded free bleeding may occur. It is a reasonable inference to make that any hæmoptysis of half an ounce, or more, probably comes from a cavity, although the latter may be much too small to be recognised by ordinary physical examination. The moment that one encounters an hæmoptysis of an ounce or more, the problem of treatment becomes all-important, because there is the likelihood of the blood being aspirated to other parts of the lung, with a corresponding spread of infection, and it must be the common experience of medical men that hæmoptysis is frequently followed by a period of fever, and not infrequently by an extension of physical signs of disease.

A third and very serious type of hæmoptysis, which is a source of great anxiety to all concerned, is one which tends to recur every few days. The problem here is one of secondary infection by septic organisms, and differs in no way from that of secondary hemorrhage in surgical conditions, except that the bleeding vessel is not accessible.

Lastly, there is the fulminating type of hemorrhage which results from the rupture of a pulmonary aneurysm, in which the patient may either bleed to death straight away, or be asphyxiated by his own blood. Fortunately, instances of this are comparatively rare and, from the point of view of treatment, have no great importance, since they are usually rapidly fatal.

Having laid down this rough clinical classification of hæmoptysis, I propose to pass on to treatment. The first class of case, exhibiting trivial initial hæmoptysis, mainly concerns us by reason of its diagnostic value. The bleeding calls for little or no special treatment, beyond the establishment of a proper regime of life. Its chief danger lies in the fact that it is liable to be unaccompanied by gross physical signs, and occasionally its true significance is overlooked. The second and third classes of cases I propose to discuss at some length.

From time immemorial the administration of morphia has occupied the premier position in the practice of a large number of doctors in the treatment of hæmoptysis. The grounds for this preference may be summed up under the following headings: (1) that it allays the anxiety of patients and friends; (2) it keeps the patient quiet and allays cough, thereby preventing further bleeding. My view is that morphia given for hæmoptysis has been responsible for more harmful results than any other drug used in the treatment of this condition. Cough occurring in association with hæmoptysis, far from being a bad thing, is one of Nature's valuable therapeutic aids. When a patient is the subject of hæmoptysis of any considerable size, it is most important that he should be able to clear his air passages of the escaped blood as quickly as possible. In the absence of a good cough reflex, blood, and infected blood at that, tends to be aspirated widely throughout the diseased lung. This blood usually spreads the tuberculous process, and if the bleeding comes from a cavity heavily contaminated with septic organisms, there is, in addition, the danger of a septic broncho-pneumonia. We are all familiar with cases which date their downward career from their first considerable hæmoptysis, which has been followed by a prolonged period of fever and signs of extension of the disease. There is usually a history of the free administration of morphia in these cases.

Those patients who are fortunate enough to escape treatment by morphia quickly clear their lungs by coughing, and little or no harm comes to them from the loss of a few ounces, or even a pint, of blood. The alleged value of morphia in allaying the apprehension of patients and friends is much overrated. If it is sensibly and quietly put to the patient that it is most important for him to get rid of the escaped blood, and that a certain amount of coughing is desirable, he ceases to worry. I know of no instruction more irksome or distressing for a patient who has blood bubbling in his bronchial tubes than to be told to inhibit his tendency to cough. Leaving out the rare tragic cases of ruptured pulmonary aneurysm, it may be laid down that all cases of hæmoptysis, even severe ones, will stop as soon as the patient has lost a moderate amount of blood, and this in spite of any form of treatment. When this stage is reached, it is all-important for the patient to get his air passages clear. The favourable results in those cases treated without morphia is reflected in the temperature chart on the days following the hæmoptysis, the majority of cases showing little or no trace of fever.

*Cough in relation to Hæmoptysis.*—To judge by the stress that is laid on the importance of morphia and absolute rest to restrain cough, one is bound to conclude that cough is almost universally regarded as a potent factor in the production of hæmoptysis. If this were a fact, hæmoptysis ought to be a common occurrence in elderly chronic bronchitics who cough violently, whereas our daily experience teaches us that such an event is rare. When it does occur, the bleeding is probably from minute branches of the bronchial artery. It will be helpful to consider briefly the mechanical effects of cough upon the chest and its contents. In the first place, the lung is suspended and maintained in a condition of expansion by the negative intrathoracic pressure. This varies around the mean point of about −8 mm. Hg. This negative pressure is due to the elastic tissue in the lung. The pressure in the pulmonary artery is about +30 mm. Hg. Between this point of highest pressure in the pulmonary circulation and the capillary bed in the lung the pressure falls rapidly, until the pressure in the capillaries is, approximately, equal to the negative intrathoracic pressure. The pulmonary capillaries are simply held open by this negative pressure. As regards the pressure in the air passages in the lung, with the glottis open the air within the lung is under atmospheric pressure. Closure of the glottis during the expulsive stage of cough raises the intrapulmonary air pressure enormously—roughly to 50 or 80 mm. Hg., according to the violence of the expulsive effort. The effect of this is to close down the capillary bed and inhibit the blood flow through the lungs; that is to say, the lungs are exsanguinated during the expulsive effort of cough, and the flow is only re-established during the succeeding inspiratory effort. It follows, then, that the expulsive effort of cough can do nothing to promote bleeding, but serves to expel escaped blood from the air passages. If the *inspiratory* effort is restrained, and I hope to show presently that it can be, cough must be an unimportant factor in the production of hæmoptysis.

It might be urged that the danger lies in the dislodgment of blood clot, the formation of which is Nature's effort to allay bleeding. Such a view, however, I hope to show, leaves out of consideration the most important factor in the production of recurrent hæmoptysis. I think it is probably within the experience of us all that serious hæmoptysis frequently occurs in patients who are resting quietly; not infrequently it rouses them from sleep. They wake up with a fit of coughing because their air passages are choked with blood, but the bleeding has, in practically every case, preceded the cough. Actually, what has happened is this. Blood-clot, which is temporarily sealing an injured vessel, becomes disorganised by the growth of septic organisms and the bleeding is really a secondary hemorrhage, such as we are familiar with in septic surgical conditions. No amount of rest or quiet can prevent the occurrence, and recurrence, of such bleeding. The only way to prevent these disasters, apart from the method I shall refer to presently, is to keep the air passages of the lung free of clot. Blood hanging on to the outside of an injured vessel in no way corks it. The point of attachment is, as a rule, but a slender pedicle which can be readily absorbed by the action of septic organisms. A minute clot fixed firmly in the orifice of an injured vessel is more effective to prevent hæmoptysis than a large clot attached by a minute pedicle, and obstructing the air way.

I propose now to discuss in some detail a method which, in my opinion, stands out as pre-eminently useful in preventing hæmoptysis. I refer to treatment by artificial pneumothorax. In the first place, it may be well to consider briefly how the method works in prevention of hemorrhage: (*a*) by compressing the lung, the blood supply through it is greatly curtailed; (*b*) the pulmonary veins and capillaries are no longer held open by the negative intrathoracic pressure—further, the introduction of even a small quantity of air into the pleural space is sufficient to prevent the inspiratory afflux of blood to the lungs, which, as I have already said, is the only important factor in a paroxysm of coughing to promote hæmoptysis; (*c*) cavities within the lung are compressed by a concentric squeeze, so that all distensile tension upon vessels spanning a cavity is abolished—this is important in the prevention of hemorrhage; (*d*) by pneumothorax the risk of aspiration of blood to other parts of the lung, and consequent extension of the tuberculous process, or widespread secondary infection, is prevented; (*e*) the ordinary nursing manipulations can be carried out freely without fear of further hemorrhage, the patient may be placed on a full diet, and the patient may be encouraged to clear his air passages of secretion as freely as possible.

*Practical Problems connected with the Induction of Artificial Pneumothorax.*—The first problem which calls for decision is to determine from which side the hemorrhage comes. In my own experience this is seldom difficult. In any case where there has been an hæmoptysis of any considerable size the signs are not usually in doubt. After hemorrhage the sound lung exhibits exaggerated breathing. On the bleeding side, in addition to weak breath-sounds, one can nearly always elicit some coarse bubbling crepitations. In the second place, the patient is usually able to give some helpful guidance as to which side is bleeding by his own subjective sensations. Thirdly, in hospital and sanatorium cases a previous knowledge of the case and X-ray records are often helpful.

Supposing, however, that there is doubt as to which side is the source of hemorrhage. I have already emphasised the fact that it is excessive inspiratory afflux of blood to the lung which is an important mechanical factor in the facilitation of bleeding, and this is abolished by even a very moderate degree of induced artificial pneumothorax. It is, therefore, quite permissible to induce a partial bilateral pneumothorax. In dealing with a unilateral induction, one would not hesitate to introduce 600 c.c. of gas into one side of the chest. It is quite permissible, therefore, to introduce 300 c.c. into each side at the same time. This is not enough to promote a serious degree of collapse from the point of view of respiratory function. It is, however, sufficient to counterbalance the effects of deep inspiratory effort, which is a main object of the proceeding. Cases so treated, however, require careful observation by X-rays at the first available opportunity, in order to estimate the degree of collapse present.

*Treatment of Hæmoptysis by Drugs.*—(*a*) Morphia, in my opinion, should have little or no place in the treatment of hæmoptysis as such. The chief use of morphia in the treatment of pulmonary tuberculosis is to allay the distress of frequent unproductive cough. This symptom, apart from the discomfort it causes the patient, is responsible for sleepless nights and continued pyrexia. While one should be very careful about administering morphia to cases with any quantity of sputum, it is frequently permissible to do so at night, and the fact that the patient has recently had slight traces of colour in the sputum I should not regard as a contra-indication for the use of some form of morphia or heroin. Morphia is, of course, particularly useful in allaying the pain of pleurisy and, by the extra rest it secures, in reducing temperature.

(*b*) Vasodilators.—One of the most useful drugs in dealing with the immediate emergency of hemorrhage is amyl nitrite. So far as I know it is not open to any objection. It does not depress the cough reflex, and I have seen hæmoptysis stopped quickly on the use of amyl nitrite, in spite of the fact that the patient has coughed freely and emptied his bronchial tubes of extravasated blood.

(*c*) Coagulants.—Various drugs reputed to produce rapid clotting are on the market, notably calcium salts, gelatine, and hæmoplastic sera. There is very little evidence that any of these have value. It is certainly a common experience of all of us that cases bleed over and over again in spite of the exhibition of large doses of these remedies. In the first place, there is no evidence that in the average patient suffering from hæmoptysis there is any defect in the coagulability of the blood.

This being the case, bleeding will always stop within a short time of its commencement. Most of us are familiar with the difficulty of obtaining more than, say, ten or twelve ounces of blood by venesection. It can, of course, be done ; but it is not easy, and the hindrance to prolonged bleeding is blood clotting. There is no evidence that artificial coagulants produce an abnormally firm clot. With recurrent hæmoptysis, it is not a question of defective coagulability of the blood but of the disintegration of the clot by septic organisms. Formation of large quantities of extravascular clot is a disaster, rather than an advantage, after an extensive hæmoptysis. We want the blood expelled, not retained. If hæmoplastic serum is to be used at all it should be by intravenous injection ; used subcutaneously it can have no value to meet an emergency of the kind under discussion.

(*d*) Mechanical Methods other than Pneumothorax.—If the principle laid down in the discussion of treatment by artificial pneumothorax is sound, viz. that one great object is to prevent excessive inspiratory movements of the lung, it stands to reason that this may be effected, though less efficiently, by firmly strapping the outside of the chest on the affected side. Strapping requires to be put on so that it well overlaps the middle line back and front, and practically the whole of the affected side of the chest must be covered with strapping. The method is, of course, only applicable when the side from which the bleeding occurs is not in any doubt, and it has the disadvantage that examination of the chest is rendered difficult and unsatisfactory.

*Distribution of the Bronchial Artery in the Lung.*—This is more extensive and better developed than is generally recognised, and the main course of the vessels follows that of the bronchial tubes. The accompanying

PLATE I.

Skiagram showing distribution of the bronchial artery in the pig. It will be noted that the main branches of the artery follow the course of the bronchi. The undulating appearance of the vessels is due to collapse of the lungs, and is an indication of the direction of pull of the elastic tissue in the lungs, which is longitudinal, and in the axes of the bronchial tubes.

[The bronchial artery was injected with barium by Dr. Bruce Perry, Colston Research Scholar, working in the University of Bristol Cardiac Research Centre at the Bristol General Hospital.]

plate shows a specimen of the bronchial artery injected in the pig. Cross sections show that the larger branches are situated in the peribronchial spaces. Only the smallest filaments lie subjacent to the mucous surface, and even this supply is by no means extensive. Herein, I think, lies the explanation of the extreme rarity of hæmoptysis of any size in chronic bronchitis. The positive expulsory effort of cough must raise the blood pressure of the aorta and arteries which come off from it. If the branches of the bronchial artery were distributed in relation to the inner surface of the bronchial tubes, somewhat profuse hæmoptysis would be a common symptom of chronic bronchitis, which it is not.

The symptom of the blood-streaked sputum is one which frequently calls for comment when cross-questioning a patient who comes complaining that he has spat up blood. The doctor usually makes a definite discrimination in his own judgment between true hæmoptysis and the spitting of sputum which is streaked with blood. If he obtains an answer that the sputum is merely blood-streaked it is taken as presumptive evidence against the existence of a tuberculous lesion in the lungs as a cause of the bleeding; this, however, without any clear conception of where the blood really comes from. Usually, on quite insufficient grounds, it is assumed that some ruptured vein in, or round, the pharynx is the cause of the bleeding. Streaks of blood which come up incorporated with sputum must of necessity have come from the air passages of the trachea or bronchi. Blood from the pharynx could not show this intimate admixture with sputum. Patients who are troubled by severe bouts of coughing are particularly prone to bring up blood-streaked sputum in the early morning, and it is precisely in these people that injury to the finer filaments of the bronchial artery distributed to the mucous surface of the bronchial tubes is to be expected.

### DISCUSSION.

DR. NEWMAN NEILD said that he thought the value of artificial pneumothorax was even greater than the lecturer had claimed. He agreed heartily as to the disadvantages of morphia, though this was the routine treatment advised even in the most recent books. He emphasised the importance of posture in keeping any retained blood out of the healthy lung. If death by drowning threatened, the patient should be inverted. Did Dr. Carleton feel sure that fever after a hæmoptysis was invariably from spread of disease, or was it possibly from absorption of extravasated blood ? He mentioned turpentine by the mouth as a drug of great value in these cases, though we did not know how it acted. Hæmoplastin, he thought, did not help.

## Figures and Tables

**Figure f1:**
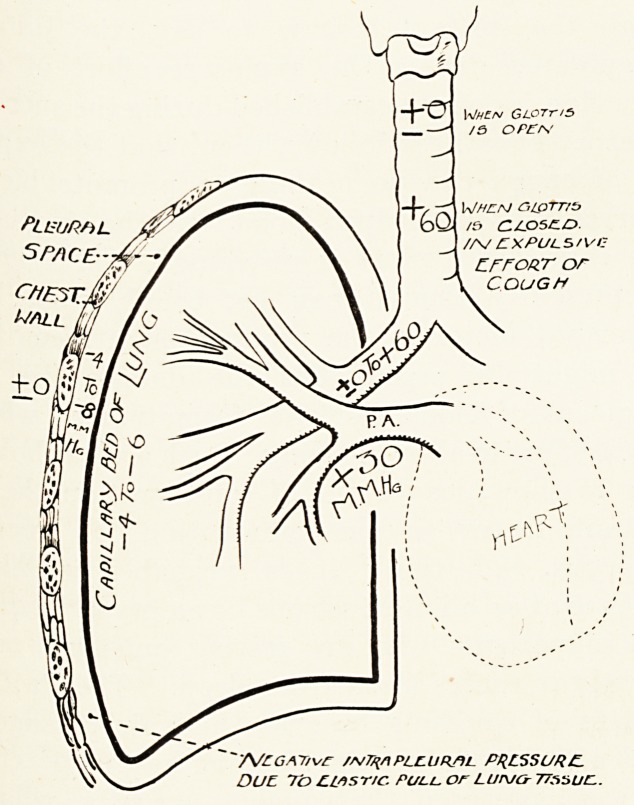


**Figure f2:**